# Participation in Conditional Cash Transfer Program During Pregnancy and Birth Weight–Related Outcomes

**DOI:** 10.1001/jamanetworkopen.2023.44691

**Published:** 2023-11-28

**Authors:** Ila R. Falcão, Rita de Cássia Ribeiro-Silva, Rosemeire L. Fiaccone, Flávia Jôse Oliveira Alves, Aline dos Santos Rocha, Naiá Ortelan, Natanael J. Silva, Poliana Rebouças, Elzo Pereira Pinto Júnior, Marcia Furquim de Almeida, Enny S. Paixao, Júlia M. Pescarini, Laura C. Rodrigues, Maria Yury Ichihara, Mauricio L. Barreto

**Affiliations:** 1 Centre for Data and Knowledge Integration for Health, Oswaldo Cruz Foundation, Salvador, Brazil; 2 School of Nutrition, Federal University of Bahia, Salvador, Brazil; 3 Department of Statistics, Federal University of Bahia, Salvador, Brazil; 4 Barcelona Institute for Global Health, Hospital Clinic, Barcelona, Spain; 5 School of Public Health, University of São Paulo, São Paulo, Brazil; 6 Epidemiology and Population Health, London School of Hygiene and Tropical Medicine, London, United Kingdom; 7 Institute of Collective Health, Federal University of Bahia, Salvador, Bahia, Brazil

## Abstract

**Importance:**

There is limited evidence of the association of conditional cash transfers, an important strategy to reduce poverty, with prevention of adverse birth-related outcomes.

**Objective:**

To investigate the association between receiving benefits from the *Bolsa Família* Program (BFP) and birth weight indicators.

**Design, Setting, and Participants:**

This cohort study used a linked data resource, the Centro de Integracao de Dados e Conhecimentos Para Saude (CIDACS) birth cohort. All live-born singleton infants born to mothers registered in the cohort between January 2012 and December 2015 were included. Each analysis was conducted for the overall population and separately by level of education, self-reported maternal race, and number of prenatal appointments. Data were analyzed from January 3 to April 24, 2023.

**Exposure:**

Live births of mothers who had received BFP until delivery (for a minimum of 9 months) were classified as exposed and compared with live births from mothers who did not receive the benefit prior to delivery.

**Main Outcomes and Measures:**

Low birth weight (LBW), birth weight in grams, and small for gestational age (SGA) were evaluated. Analytical methods used included propensity score estimation, kernel matching, and weighted logistic and linear regressions. Race categories included Parda, which translates from Portuguese as “brown” and is used to denote individuals whose racial background is predominantly Black and those with multiracial or multiethnic ancestry, including European, African, and Indigenous origins.

**Results:**

A total of 4 277 523 live births (2 085 737 females [48.8%]; 15 207 among Asian [0.4%], 334 225 among Black [7.8%], 29 115 among Indigenous [0.7%], 2 588 363 among Parda [60.5%], and 1 310 613 among White [30.6%] mothers) were assessed. BFP was associated with an increase of 17.76 g (95% CI, 16.52-19.01 g) in birth weight. Beneficiaries had an 11% lower chance of LBW (odds ratio [OR], 0.89; 95% CI, 0.88-0.90). BFP was associated with a greater decrease in odds of LBW among subgroups of mothers who attended fewer than 7 appointments (OR, 0.85; 95% CI, 0.84-0.87), were Indigenous (OR, 0.73; 95% CI, 0.61-0.88), and had 3 or less years of education (OR, 0.76; 95% CI, 0.72-0.81). There was no association between BFP and SGA, except among less educated mothers, who had a reduced risk of SGA (OR, 0.83; 95% CI, 0.79-0.88).

**Conclusions and Relevance:**

This study found that BFP was associated with increased birth weight and reduced odds of LBW, with a greater decrease in odds of LBW among higher-risk groups. These findings suggest the importance of maintaining financial support for mothers at increased risk of birth weight–related outcomes.

## Key Points

**Question:**

Is the *Bolsa Família* Program (BFP) associated with improved birth weight–related outcomes?

**Findings:**

This cohort study of 4 277 523 live births found that BFP participation was associated with a reduction in low birth weight and an increase in birth weight. Outcomes were more pronounced among groups at increased risk, with a greater decrease in odds of low birth weight found among mothers with fewer years of education and indigenous mothers.

**Meaning:**

These findings suggest that BFP may be associated with improved birth weight–related outcomes and decreased birth weight inequalities.

## Teaser

This cohort study investigates the association of participation in the *Bolsa Família* cash transfer program with birth weight–related outcomes.

## Introduction

Birth weight and gestational age are crucial indicators of adverse health outcomes at birth.^1,2,3,4,5^ Low birth weight (LBW) is a marker of risk among newborns, with short- and long-term consequences, and is therefore a concern, particularly in low- and middle-income countries.^6^ In Latin America and the Caribbean, 8.7% of live births are considered LBW^6^ and 12.5% are considered small for gestational age (SGA).^7^ The prevalence of LBW in Brazil is approximately 8.7%,^8^ and this has not significantly decreased in the last 15 years.^6^ SGA births^9^ correspond to 7.8% of births in the 100 Million Cohort.^10^

In low- and middle-income countries, socioeconomic factors, including education, income, self-reported race, and access to prenatal care, are associated with birth weight and SGA.^10,11,12,13,14,15,16,17,18,19^ Conditional cash transfer (CCT) programs have emerged in Latin America beginning in the 1990s as a strategy for social protection and poverty reduction.^20,21^ Complementary to unconditional cash transfer programs (UCTs), which provide only monetary transfers, CCTs incorporate the fulfillment of conditionalities (typically, adherence to a health and education agenda) as a requirement for continued receipt.^20,21^ Thus, CCTs may be associated with reductions in barriers to accessing services, increased income and food access, and, consequently, promotion of maternal and child health.^22,23,24,25^

CCTs have been associated with lower child^26^ and maternal mortality,^27^ improvements in child nutrition and health,^28,29^ preventive behavior, and an increase in the use of health services.^23^ Despite this potential to stimulate positive health-related behaviors, a recent literature review indicated that due to the CCT health conditionality component characteristic, there was a lack of understanding about whether cash transfers are more effective in specific subgroups of the population than others.^30^

The *Bolsa Família* Program (BFP) is one of the world’s pioneering CCTs. It has more than 13 million beneficiary families per year.^31^ Although Brazil was one of the pioneers in implementing CCTs in Latin America and there has been some evaluation of the association of this program with child health,^22,23,24,26^ there is still a lack of evidence to support an association of the BFP with birth weight indicators.

Our objective was to estimate the effectiveness of PBF, focusing on its potential association with a decreased likelihood of LBW and SGA, as well as improved birth weight (in grams). It is recognized that the association of BFP with birth weight indicators may vary by population subgroup.

## Methods

### Ethical Considerations

The Research Ethics Committee of the Institute of Collective Health, Federal University of Bahia approved the protocol for this cohort study and waived informed consent because this study uses electronic data without any personally identifiable information. The Reporting of Studies Conducted Using Observational Routinely Collected Health Data (RECORD) statement has been followed.

### Study Population

The eligible study population consisted of children from live births in the Centro de Integracao de Dados e Conhecimentos Para Saude (CIDACS) Birth Cohort^32^ from 2012 to 2015 among mothers aged 10 to 49 years who were registered on CadÚnico (the Brazilian national social program register) at any time from 2004 to 2015 (Figure). Births before the mother entered the cohort, births before the study period, and individuals with inconsistencies in variables (eg, mother’s age) and missing data on outcomes were considered ineligible for the study (eFigure 1 and eAppendix 1 in Supplement 1). Our selection was limited to births that occurred between 2012 and 2015 due to a change to birth certificates in 2011. The live birth certificate includes crucial variables for our study, such as gestational age in weeks, place of birth (hospital, maternity center, and other), and number of prenatal consultations (as a quantitative variable). Exclusion criteria were (1) live births without fetal viability^33,34^ (birth weight <500 g or born before 22 gestational weeks) and (2) multiple births and newborns with congenital anomalies (given that these conditions are associated with adverse birth weight indicators^35^).

**Figure.**
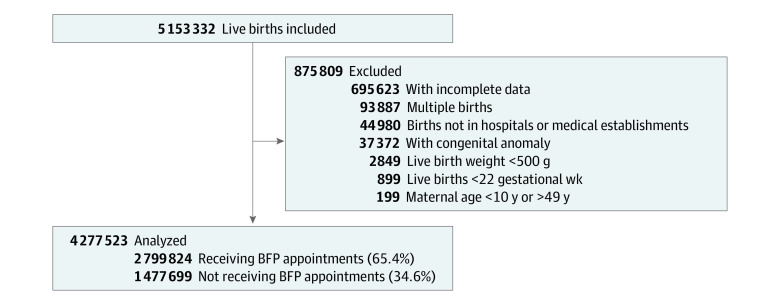
Study Flowchart BFP indicates *Bolsa Família* Program.

### Exposure

Live births were classified as being exposed to BFP if the mother started receiving BFP at any time during the cohort period, considering an exposure window of at least the estimated period of a complete pregnancy (9 months), without interruptions. Mothers who discontinued receipt were not considered in the analysis. Newborns of mothers who did not receive BFP at any time before delivery were classified as unexposed. BFP eligibility criteria are CadÚnico registered family per capita income and family composition (such as the presence of children, adolescents, and pregnant individuals). Families with a monthly per capita income of up to R $89.00 (income cutoff point for 2019; equivalent to US $22.00) are considered extremely low income and eligible, independent of their composition.^36^ Low-income families (per capita income between R $89.01 [income cutoff point for 2019; equivalent to US $22.00] and R $178.00 [income cutoff point for 2019; equivalent to US $44.00]) are eligible for the BFP if they include at least 1 individual from a priority group, such as pregnant individuals, breastfeeding mothers, children, or children or adolescents aged 0 to 17 years.^36^ Ideally, cash payments are directed toward women, contingent on the fulfillment of specific program requirements (conditionalities).^35,36^ These criteria encompass the necessity for consistent school attendance and use of health care services throughout childhood (including maintaining an up-to-date vaccination schedule), during pregnancy (prenatal consultations), and in the postpartum period.^37^ Further details on eligibility criteria and program characteristics are described in eAppendix 2 in Supplement 1.

### Study Design and Data Sources

This is a retrospective cohort. The study considered socioeconomic and demographic data from the 100 Million Brazilian Cohort,^38^ linked to the Live Birth Information System (SINASC) from January 1, 2004, to December 31, 2015. The cohort database contains records of 114 001 661 individuals (40 542 929 families) with low income eligible for social assistance programs via CadÚnico. This linkage constitutes the CIDACS Birth Cohort (a subset of the 100 Million Brazilian Cohort).^32^ All data sets were evaluated with deidentified, linked data (eAppendix 3 in Supplement 1). More information about the databases and linkage is presented in eAppendix 3 and eFigures 2 and 3 in Supplement 1. Socioeconomic and housing information at the individual level is taken from the cohort baseline, and characteristics of the mother and newborn are taken from SINASC records. The variable related to maternal race was derived from the CadÚnico database, collected through self-report as Asian, Black, Indigenous, Parda, or White. The term Parda translates from Portuguese as “brown” and is used to denote individuals whose racial background is predominantly Black and those with multiracial or multiethnic ancestry, including European, African, and Indigenous origins.

### Outcomes

Our outcomes were birth weight categorized as LBW (<2500 g) and non-LBW (2500 g to <4000 g; reference group), birth weight in grams, and SGA (<10th percentile of weight for gestational age according to sex) and appropriate for gestational age (reference group; 10th-90th percentile). Newborn size was defined by sex-specific curves corresponding to single live births as established by the International Fetal and Newborn Growth Consortium for the 21st Century (INTERGROWTH-21st) Consortium^39^ to classify weight at gestational age (24/0 to 42/0 gestational weeks).

### Statistical Analysis

We estimated the association of BFP with birth-related indicators using propensity score (PS)–based methods. Analyses were described in detail in the research protocol.^40^ PS estimation was performed using complete data (main analysis). The descriptive analysis based on missing data is available in eTables 1-4 and eAppendix 4 in Supplement 1. Additionally, we performed the PS estimation incorporating a missing data category (category 7 = missing data) (eTable 5 and eAppendix 5 in Supplement 1). Our analysis involved a PS estimation through a logistic model to estimate the probability of receiving BFP based on confounding variables observed (eAppendix 5, eFigure 4, and eTables 6-7 in Supplement 1) and year of cohort entry. BFP beneficiary and nonbeneficiary individual weights were estimated from the PS through kernel-based matching.^41^ A 2-sided *P* value < .05 indicated statistical significance.

To estimate the association of BFP with LBW and SGA, we used logistic models weighted and adjusted for the following risk factors (categorical variables): gestational age, sex of live-born infant, maternal age at birth, and type of delivery. Adjusted risk ratio was calculated using the δ method to verify discrepancies in the odds ratio (OR) (eTables 8-9 and eAppendix 6 in Supplement 1). A linear model was used to estimate the association of BFP with birth weight (continuous, by 1-g increase in weight) weighted and adjusted by gestational age in weeks, maternal age at birth in years, sex of the live-born infant, and type of delivery (eTable 6 in Supplement 1). We used inverse probability of treatment weighting (IPTW)^42^ as an alternative approach (eTable 10 and eAppendix 7 in Supplement 1) to estimate the association between BFP participation and birth weight indicators. Analyses were performed using Stata statistical software version 16 (StataCorp). Data were analyzed from January 3 to April 24, 2023.

We aimed to explore BFP association with birth indicators across subgroups according to self-reported maternal race (Asian, Black, Indigenous, Parda, and White), educational level (≥8, 4-7, and ≤3 years), and attendance at prenatal appointments (<7 and ≥7 appointments). All PSs were estimated separately for each population subgroup, with the variable defining the subgroup excluded from the calculation. Similarly, kernel-weighted logistic and linear models were calculated overall and separately within each population subgroup (eTable 11 and eAppendix 8 in Supplement 1). Given well-established associations of socioeconomic disadvantage and racial disparities with maternal health and use of health care services, we conducted subgroup analyses based on attending prenatal appointments, maternal education, and self-reported race.^11,12,13,23,26,30,43,44^ Additionally, we evaluated unadjusted associations of BFP, maternal education, and self-reported race with attendance of prenatal appointments (eTable 14 and eAppendix 9 in Supplement 1).

Furthermore, we examined the association of BFP with birth outcomes among multiparous mothers, while also accounting for characteristics of their prior pregnancies through use of weighted and adjusted models (eTable 12 in Supplement 1). Additionally, we investigated the association between BFP and birth outcomes based on PS quintile (eTable 13 in Supplement 1).

## Results

Of 5 153 332 eligible live births to mothers entering the cohort between 2004 and 2015, 4 973 146 live births were initially included in the study (Figure). Of these, 695 623 births (14.0%) had incomplete data in at least 1 variable used to calculate the PS. Therefore, our analysis included 4 277 523 live births (2 085 737 females [48.8%]; 15 207 among Asian [0.4%], 334 225 among Black [7.8%], 29 115 among Indigenous [0.7%], 2 588 363 among Parda [60.5%], and 1 310 613 among White [30.6%] mothers) from 2012 to 2015.

Approximately one-third of live births (1 477 699 births [34.6%]) were to nonbeneficiary mothers, and 2 799 824 births (65.4%) were to mothers who received BFP. Mean (SD) and median (IQR) birth weight among mothers who received BFP (3228.2 [521.4] g and 3240.0 [2940.0-3550.0] g, respectively) were higher than among mothers who did not receive BFP (3207.0 [524.7] g and 3225.0 [2925.0-3530.0] g, respectively) (Table 1). Occurrence of LBW was lower among births from beneficiaries than nonbeneficiaries (186 184 births [6.6%] vs 104 322 births [7.1%]), and SGA was higher among births from beneficiaries than nonbeneficiaries (216 678 of 2 253 931 births with data [9.6%] vs 107 718 of 222 037 births with data [8.8%]) (Table 1).

**Table 1.** Birth Weight Indicators of Live-Born Children

Outcome	Live births, No. (%) (N = 4 277 523)								
	Overall			≥7 Appointments^a^			<7 Appointments^a^		
	BFP (n = 2 799 824)	No BFP (n = 1 477 699)	Total (n = 4 277 523)	BFP (n = 1 566 956)	No BFP (n = 983 824)	Total (n = 2 550 780)	BFP (n = 1 153 015)	No BFP (n = 456 922)	Total (n = 1 609 937)
Birth weight, g									
Mean (SD)	3228.2 (521.4)	3207 (524.7)	3220.9 (522.6)	3279.3 (475.7)	3253.9 (473.8)	3269.5 (475.1)	3164.6 (565.6)	3113.6 (602.4)	3150.1 (576.7)
Median (IQR)	3240.0 (2940.0-3550.0)	3225.0 (2925.0-3530.0)	3235.0 (2930.0-3545.0)	3275.0 (2990.0-3580.0)	3250.0 (2970.0-3550.0)	3265.0 (2980.0-3565.0)	3200.0 (2870.0-3510.0)	3165.0 (2830.0-3480.0)	3190.0 (2860.0-3500.0)
Range	500.0-6999.0	500.0-6985.0	500.0-6999.0	500.0-6999.0	500.0-6985.0	500.0-6999.0	500.0-6999.0	500.0-6970.0	500.0-6999.0
LBW^b^									
No	2 613 640 (93.4)	1 373 377 (92.9)	3 987 017 (93.2)	1 497 360 (95.6)	936 104 (95.2)	2 433 464 (95.4)	1 044 792 (90.6)	404 479 (88.5)	1 449 271 (90.0)
Yes	186 184 (6.6)	104 322 (7.1)	290 506 (6.8)	69 596 (4.4)	47 720 (4.8)	117 316 (4.6)	108 223 (9.4)	52 443 (11.5)	160 666 (10.0)
SGA^c^									
Total with data, No.^d^	2 253 931	222 037	3 475 968	1 278 061	819 068	2 097 129	927 913	379 221	1 307 134
No	2 037 253 (90.4)	1 114 319 (91.2)	3 151 572 (90.7)	1 163 469 (91.0)	752 131 (91.8)	1 915 600 (91.3)	832 080 (89.7)	341 520 (90.1)	1 173 600 (89.8)
Yes	216 678 (9.6)	107 718 (8.8)	324 396 (9.3)	114 592 (9.0)	66 937 (8.2)	181 529 (8.7)	95 833 (10.3)	37 701 (9.9)	133 534 (10.2)

Abbreviation: SGA, small for gestational age.

^a^ Appointments categorized by the median.

^b^ LBW was defined as birth weight less than 2500 g and not LBW as birth weight 2500 g to less than 4000 g.

^c^ SGA was defined as weight for gestational age at birth less than the 10th percentile of weight for gestational age according to sex, and not SGA (ie, appropriate for gestational age) was defined as weight for gestational age at birth in the 10th to 90th percentile.

^d^ To calculate SGA populations, the International Fetal and Newborn Growth Consortium for the 21st Century Consortium considers only infants born between 24 and 42 gestational weeks. Live births that were not within this range were considered missing.

According to PS variables, differences between beneficiaries and nonbeneficiaries were minimized after weighting (eg, mothers with ≥8 years of schooling: 63.1% vs 63.0%; difference in proportion, 0.1 percentage points) (Table 2). BFP was associated with an 11% lower LBW risk (OR, 0.89; 95% CI, 0.88-0.90) (Table 3). Participation in BFP was associated with an increase of 17.76 g (95% CI, 16.52-19.01 g) in birth weight. However, there was no association between BFP participation and odds of SGA (OR, 0.99; 95% CI, 0.98-1.00). A robustness test using the IPTW method showed similar results (eTable 10 in Supplement 1).

**Table 2.** Variables With Complete Data Used for PS

PS variable	Mothers of live births^a^																	
	Overall						≥7 Appointments						<7 Appointments					
	Unweighted			Weighted			Unweighted			Weighted			Unweighted			Weighted		
	Proportion, %		Diff^c^	Proportion, %		Diff^c^	Proportion, %		Diff^c^	Proportion, %		Diff^c^	Proportion, %		Diff^c^	Proportion, %		Diff^c^
	No BFP^b^	BFP^b^		No BFP^b^	BFP^b^		No BFP^b^	BFP^b^		No BFP^b^	BFP^b^		No BFP^b^	BFP^b^		No BFP^b^	BFP^b^	
Sociodemographic characteristic																		
Level of education, y																		
≥8	79.5	63.0	−16.5	63.0	63.1	0.1	82.8	67.4	−15.4	80.6	67.4	−13.2	73.2	57.5	−15.6	69.2	57.6	−11.7
4-7	18.2	30.9	12.8	31.4	30.9	−0.5	15.4	27.7	12.3	17.0	27.7	10.7	23.5	35.0	11.5	26.3	35.0	8.7
≤3	2.3	6.1	3.7	5.7	6.0	0.3	1.8	4.9	3.1	2.3	4.9	2.5	3.3	7.5	4.2	4.5	7.4	3.0
Race^d^																		
Asian	0.4	0.3	−0.1	0.3	0.3	0.0	0.4	0.3	0.0	0.3	0.3	0.0	0.4	0.3	−0.1	0.3	0.3	0.0
Black	6.7	8.4	1.6	8.5	8.4	−0.1	6.3	8.0	1.7	8.0	8.0	0.0	7.5	8.8	1.3	8.8	8.8	0.0
Indigenous	0.3	0.9	0.6	0.8	0.9	0.1	0.2	0.6	0.4	0.5	0.6	0.1	0.4	1.3	0.9	1.1	1.2	0.1
Parda^e^	52.4	64.8	12.4	65.0	64.8	−0.2	49.4	61.8	12.4	62.0	61.9	−0.1	58.7	68.9	10.2	69.2	68.9	−0.3
White	40.2	25.6	−14.6	25.4	25.6	0.2	43.6	29.2	−14.4	29.2	29.2	0.0	33.0	20.7	−12.3	20.6	20.7	0.1
Marital status																		
Partner	54.6	51.4	−3.3	50.2	51.4	1.1	58.1	54.3	−3.8	53.7	54.3	0.6	48.0	48.2	0.2	47.6	48.2	0.6
No partner	45.4	48.6	3.3	49.8	48.6	−1.1	41.9	45.7	3.8	46.3	45.7	−0.6	52.0	51.8	−0.2	52.4	51.8	−0.6
Housing characteristic																		
Area of residency																		
Urban	84.0	71.5	−12.5	72.2	71.6	−0.6	84.1	72.3	−11.8	72.7	72.3	−0.4	83.6	70.3	−13.3	70.9	70.4	−0.5
Rural	16.0	28.5	12.5	27.8	28.4	0.6	15.9	27.7	11.8	27.3	27.7	0.4	16.4	29.7	13.3	29.1	29.6	0.5
Construction materials																		
Brick	80.5	68.7	−11.8	68.8	68.8	0.0	81.7	72.1	−9.7	72.1	72.1	0.0	77.8	64.1	−13.7	64.1	64.2	0.1
Wood, or other	19.5	31.3	11.8	31.2	31.2	0.0	18.3	27.9	9.7	27.9	27.9	0.0	22.2	35.9	13.7	35.9	35.8	−0.1
Water supply																		
Public network	78.7	65.1	−13.6	65.5	65.2	−0.3	80.1	67.5	−12.6	67.7	67.5	−0.2	75.8	61.9	−13.9	62.0	62.0	0.0
Well, or other	21.3	34.9	13.6	34.5	34.8	0.3	19.9	32.5	12.6	32.3	32.5	0.2	24.2	38.1	13.9	38.0	38.0	0.0
Home with electricity meter																		
Yes	89.5	79.1	−10.4	78.9	79.2	0.3	91.0	82.1	−8.9	82.0	82.2	0.1	86.3	75.0	−11.3	74.8	75.1	0.3
No	10.5	20.9	10.4	21.1	20.8	−0.3	9.0	17.9	8.9	18.0	17.8	−0.1	13.7	25.0	11.3	25.2	24.9	−0.3
Waste collection																		
Collected	84.4	68.6	−15.8	69.1	68.7	−0.3	85.2	70.6	−14.7	71.1	70.6	−0.4	82.7	66.0	−16.8	66.2	66.1	−0.2
Burned, buried, or other	15.6	31.4	15.8	30.9	31.3	0.3	14.8	29.4	14.7	28.9	29.4	0.4	17.3	34.0	16.8	33.8	33.9	0.2
Sanitation system																		
Public network	52.6	37.6	−15.0	37.9	37.6	−0.2	54.4	40.0	−14.4	40.2	40.0	−0.1	48.4	34.1	−14.3	34.4	34.1	−0.3
Septic tank or other	47.4	62.4	15.0	62.1	62.4	0.2	45.6	60.0	14.4	59.8	60.0	0.1	51.6	65.9	14.3	65.6	65.9	0.3
Overcrowding (>2 inhabitants/room)																		
No	93.6	87.0	−6.7	86.8	87.1	0.2	94.6	88.9	−5.7	88.8	89.0	0.2	91.7	84.5	−7.2	84.5	84.6	0.1
Yes	6.4	13.0	6.7	13.2	12.9	−0.2	5.4	11.1	5.7	11.2	11.0	−0.2	8.3	15.5	7.2	15.5	15.4	−0.1
Year of entry into cohort baseline																		
2004-2005	12.6	11.1	−1.4	13.0	11.1	−1.9	12.8	11.3	−1.5	13.2	11.3	−1.9	11.9	10.8	−1.1	13.0	10.7	−2.3
2006-2007	53.6	64.8	11.2	64.4	64.8	0.5	53.5	64.9	11.4	64.8	64.9	0.1	53.7	64.7	11.0	64.6	64.7	0.1
2008-2009	8.0	12.4	4.4	10.5	12.4	1.9	7.8	12.0	4.2	10.0	12.0	2.0	8.4	12.9	4.5	10.6	12.9	2.3
2010-2011	6.5	6.8	0.3	5.8	6.8	1.0	6.6	6.8	0.2	5.8	6.8	1.0	6.4	6.9	0.4	5.6	6.8	1.3
2012-2015	19.3	4.9	−14.4	6.3	4.9	−1.5	19.2	5.0	−14.2	6.1	5.0	−1.1	19.6	4.8	−14.9	6.2	4.8	−1.4

Abbreviations: BFP, *Bolsa Família* Program; diff, difference; PS, propensity score.

^a^ Populations and percentages are given before and after kernel weighting.

^b^ Overall, there were 4 277 523 unweighted births total, including 1 477 699 births in the non-BFP and 2 799 824 births in the BFP group, and 4 270 037 weighted births total, including 1 476 408 births in the non-BFP and 2 793 629 births in the BFP group. Among mothers with 7 or more appointments, there were 2 550 780 unweighted births total, including 983 824 births in the non-BFP and 1 566 956 births in the BFP group, and 2 548 190 weighted births total, including 983 330 births in the non-BFP and 1 564 860 births in the BFP group. Among mothers with fewer than 7 appointments, there were 1 609 937 unweighted births total, including 456 922 births in the non-BFP and 1 153 015 births in the BFP group, and 1 606 877 weighted births total, including 456 446 births in the non-BFP and 1 150 431 births in the BFP group.

^b^ The difference in proportion of each category is given between BFP beneficiaries and nonbeneficiaries. Units are percentage points.

^d^ Race was self-reported.

^e^ Parda, which translates from Portuguese as “brown,” is used to denote individuals whose racial background is predominantly Black and those with multiracial or multiethnic ancestry, including European, African, and Indigenous origins.

**Table 3.** Association of *Bolsa Família* Participation With Birth Weight Indicators

Model	Adjusted outcome (95% CI)	Robust SE	*P* value	Live births included, No.
Model 1: LBW, OR^a^^,^^b^	0.89 (0.88-0.90)	0.005	<.001	4 232 863
Model 2: SGA, OR^a^^,^^c^	0.99 (0.98-1.00)	0.005	.08	3 464 938
Model 3: birth weight, β^d^	17.76 (16.52-19.01)	0.638	<.001	4 232 863

Abbreviations: LBW, low birth weight; OR, odds ratio; SE, standard error; SGA, small for gestational age.

^a^ In logistic regression results, the analysis was kernel weighted and adjusted for gestational age, sex of the live-born child, mother’s age at birth, and type of delivery.

^b^ LBW was defined as birth weight less than 2500 g, and not LBW was defined as birth weight 2500 g to less than 4000 g.

^c^ SGA was defined as weight for gestational age at birth less than the 10th percentile of weight for gestational age according to sex, and not SGA (ie, appropriate for gestational age) was defined as weight for gestational age at birth in the 10th to 90th percentile.

^d^ In linear regression results, the analysis was kernel weighted and adjusted for gestational age, sex of the live-born child, mother’s age at birth, and type of delivery.

Considering the frequency of prenatal care appointments attended, BFP participation was associated with a greater reduction in odds of LBW (OR, 0.85; 95% CI, 0.84-0.87) and greater increase in birth weight (β = 25.09 g; 95% CI, 22.91-27.26 g) among mothers who attended fewer than 7 appointments (Table 4). Estimates for LBW varied from a 7% reduction in odds for live births among White mothers who received BFP (OR, 0.93; 95% CI, 0.91-0.94) to a 27% reduction (OR, 0.73; 95% CI, 0.61-0.88) for Indigenous mothers who received BFP (Table 4). BFP was associated with a particularly large reduction in odds of SGA among Indigenous mothers (OR, 0.79; 95% CI, 0.67-0.92). Additionally, BFP was associated with a greater reduction in odds of LBW (OR, 0.76; 95% CI, 0.72-0.81) and SGA (OR, 0.83; 95% CI, 0.79-0.88) and in birth weight (β = 56.02 g; 95% CI, 48.03-64.00 g) in live births of mothers with less than 3 years of formal education. The analysis for a specific subpopulation of multiparous mothers indicated an association between BFP and LBW (OR, 0.95; 95% CI, 0.92-0.98) (eTable 12 in Supplement 1). Considering the analysis by PS quintiles, we observed that BFP was associated with a greater decrease in LBW odds and a greater increase in birth weight as the higher quintile was evaluated (eTable 13 in Supplement 1). In the fifth quintile, BFP was associated with a 25% lower chance of LBW (OR, 0.85; 95% CI, 0.82-0.87), 6% lower odds of SGA (OR, 0.94; 95% CI, 0.91-0.96), and an increase in birth weight of 27.08 g (95% CI, 23.65-30.50 g).

**Table 4.** Association of *Bolsa Família* Participation With Birth Weight Indicators by Subgroup

Subgroup	LBW^a^				SGA^b^				Birth weight			
	OR (95% CI)^c^	Robust SE	*P* value	Live births, included, No.	OR (95% CI)^c^	Robust SE	*P* value	No.	β (95% CI), g^c^	Robust SE	*P* value	Live births included, No.
Prenatal appointments, No.												
Model 7: ≥7	0.93 (0.91-0.94)	0.008	<.001	2 542 051	1.02 (1.00-1.03)	0.007	.008	2 089 585	13.22 (11.71-14.74)	0.772	<.001	2 542 051
Model 8: <7	0.85 (0.84-0.87)	0.007	<.001	1 601 658	0.97 (0.96-0.99)	0.008	.001	1 299 972	25.09 (22.91-27.26)	1.109	<.001	1 601 658
Self-reported maternal race												
Model 9: Asian	0.83 (0.66-1.03)	0.092	.09	14 780	0.99 (0.83-1.19)	0.091	.92	12 088	13.96 (−7.05-34.96)	10.717	.19	14 780
Model 10: Black	0.86 (0.83-0.89)	0.017	<.001	329 995	0.94 (0.91-0.97)	0.016	.001	272 129	24.23 (19.66-28.80)	2.331	<.001	329 995
Model 11: Indigenous	0.73 (0.61-0.88)	0.069	.001	28 369	0.79 (0.67-0.92)	0.064	.003	22 258	37.40 (16.21-58.60)	10.813	.001	28 369
Model 12: Parda^d^	0.88 (0.87-0.90)	0.007	<.001	2 566 408	0.99 (0.98-1.00)	0.007	.08	2 087 464	18.76 (17.13-20.40)	0.835	<.001	2 566 408
Model 13: White	0.93 (0.91-0.94)	0.009	<.001	1 293 632	1.04 (1.02-1.06)	0.009	<.001	1 071 086	10.51 (8.54-12.49)	1.009	<.001	1 293 632
Maternal level of education, y												
Model 14: ≥8	0.91 (0.90-0.93)	0.006	<.001	2 913 202	1.03 (1.02-1.04)	0.006	<.001	2 402 501	10.41 (9.10-11.72)	0.670	<.001	2 913 202
Model 15: 4-7	0.88 (0.86-0.90)	0.010	<.001	1 120 062	0.96 (0.94-0.98)	0.010	<.001	906 413	24.14 (21.51-26.76)	1.339	<.001	1 120 062
Model 16: ≤3	0.76 (0.72-0.81)	0.024	<.001	199 483	0.83 (0.79-0.88)	0.023	<.001	155 603	56.02 (48.03-64.00)	4.074	<.001	199 483

Abbreviations: LBW, low birth weight; SGA, small for gestational age.

^a^ LBW was defined as birth weight less than 2500 g, and not LBW was defined as birth weight 2500 g to less than 4000 g.

^b^ SGA was defined as weight for gestational age at birth less than the 10th percentile of weight for gestational age according to sex, and not SGA (ie, appropriate for gestational age) was defined as weight for gestational age at birth in the 10th to 90th percentile.

^c^ Analytical steps (propensity score estimation, kernel matching, and weighted regression) were conducted separately within each level of education, self-reported maternal race, and number of appointments. The analysis was kernel weighted and adjusted for gestational age, sex of the live-born child, mother’s age at birth, and type of delivery. LBW and SGA analyses used logistic regression, and the birth weight analysis used linear regression.

^d^ Parda, which translates from Portuguese as “brown,” is used to denote individuals whose racial background is predominantly Black and those with multiracial or multiethnic ancestry, including European, African, and Indigenous origins.

## Discussion

In this cohort study, we found that BFP participation was associated with reduced chances of LBW and an increase in birth weight in grams. BFP participation was associated with a greater decrease in odds of LBW and increase in birth weight in grams among higher-risk population subgroups classified in our study: mothers who attended fewer than 7 antenatal care appointments; were Black, Indigenous, or Parda; and less educated (≤3 years of formal education). An association between BFP participation and decreased odds of SGA was found among Indigenous mothers and those with less education.

Our findings are consistent with those of a previous study that examined 100 Million Brazilian Cohort data and other studies that evaluated the effect of CCTs on birth weight.^45,46,47^ However, we also explored the association of BFP with outcomes in pregnant individuals from different social and ethnic subgroups, showing greater changes in outcomes among the highest-risk groups. In addition, use of information on previous childbirths enabled adjustment for birth intervals, previous LBW, and previous prematurity.^19,48^ Although CCTs have an association with an increased interval between births,^49^ the association with beneficiary fertility among mothers is controversial.^50^ The first pregnancy and grand multiparity are risk factors for LBW and SGA.^10,19^

The magnitude of outcomes associated with other CCTs and UCTs has varied by program characteristic. A study of the effectiveness of the Oportunidades program, a CCT implemented in Mexico, demonstrated a 127-g increase in mean weight at birth among beneficiary children and a 4.6% lower prevalence of LBW in this group.^45^ A randomized study conducted in rural villages in Togo, West Africa, found that receiving a UCT reduced the chance of having a baby with LBW (adjusted OR, 0.29; CI 95%, 0.10-0.82).^46^ In Colombia, a study on the Familias en Acción program showed a 578-g increase in birth weight in urban treatment locations.^47^ Increased birth weight in the US Food Stamp Program (currently known as the Supplemental Nutrition Assistance Program) provides further evidence that prenatal nutritional intake may play a role in child birth outcomes.^51^ In the US, Special Supplemental Nutrition Program for Women, Infants, and Children (WIC) services have also been associated with reduced LBW and increased birth weight in grams, especially among subgroups of Black women and those with late prenatal care or no prenatal consultation.^52^

Cash transfer strategies are also implemented in high-income countries.^53,54^ In the US, poverty relief during the prenatal period (an income tax credit) was associated with an increase in birth weight of 15.7g (12.5 g when adjusted for smoking).^53^ In retrospective cohort studies, cash transfers during the prenatal period provided to women with lower incomes who were residents in a municipality of Canada (through the Healthy Baby Prenatal Benefit UCT) were associated with a 26%^55^ and 29%^56^ lower risk of LBW.

To the best of our knowledge, this is the first study to evaluate the association of CCT with weight standards at birth by gestational age in a high-risk Brazilian population. In a study in Canada, the only study found that assessed the association of a CT (specifically, a UCT) with SGA, an association was found with a decrease in SGA births (adjusted risk ratio, 0.90; 95% CI, 0.81-0.99).^55^

The difference between CT designs may explain the variability reported in estimates (heterogeneity of findings). We may consider 2 hypotheses for the mechanisms behind the association of CCTs with birth weight outcomes. First, CTs enable the family to diversify the food purchased (consuming more vegetables, fruit, and meat, which are sources of minerals and vitamins), which is associated with family food security indicators,^46,47,56^ psychosocial health,^57^ increased social capital, and female decision-making power.^57,58^ The second is the association of conditionalities with outcomes and the benefits provided by integrated, health-related actions.^23,47,59,60,61^

Despite the significant increase in attending prenatal appointments in Brazil between 2000 and 2015, inequality remains pronounced, particularly among Black and Indigenous women and those with a lower level of education.^62^ These groups include individuals experiencing more deprivation with greater difficulty in accessing this service. Our investigation found associations between BFP and increased birth weight and decreased odds of LBW within specific subgroups. These subgroups included mothers who attended fewer prenatal appointments; those who were Black, of mixed race, and Indigenous; and those with lower levels of education. Furthermore, our study found that BFP beneficiaries had lower odds of SGA only in subgroups of Indigenous mothers and those with lower education levels. These results suggest that beneficiaries and nonbeneficiaries may be more homogeneous in relation to characteristics not observed in subgroups of mothers at higher risk. The group with a lower number of prenatal consultations likely consisted of individuals who faced greater challenges in accessing this service, particularly those with lower socioeconomic status. Education was the only variable that was not well-balanced between groups. Therefore, disparities persisted, with a higher percentage of mothers in the group with fewer consultations having low levels of education. These findings are consistent with those of a recent review on CCTs and child health in low- and middle-income countries showing that these programs exhibited considerable heterogeneity among subgroups by socioeconomic status indicator.^30^

LBW, as a result of poverty, can contribute to worse health status over time and consequently maintain inequality from generation to generation.^53^ The difficulty of reducing birth weight–related outcomes indicates the need to intensify policies with this focus.^6^ Thus, there is a need to strengthen social, redistributive, and health policies that act on the negative consequences of inequalities, seeking to minimize their effects on health, striving for food and nutritional security, prenatal care, and assistance during labor.^12^

### Strengths and Limitations

This study used PS-based approaches to evaluate the association of BFP with maternal-child health results in a population of low-income and extremely low-income Brazilian families. The study followed a previously defined and published research protocol,^40^ providing data analysis transparency and greater result comparability. Several strengths can be highlighted in this study. The population-level database encompasses a wide range of socioeconomic variables at family and personal levels and a variety of risk factors, which are rarely available in administrative data. A robust analytical approach using kernel-based PS weighting and IPTW was used to account for observed confounding factors in the study. Beneficiary and nonbeneficiary groups were well-balanced for covariate distributions.

Several limitations should also be considered. Receiving BFP is not a random attribution but the result of a self-selection process by families. A BFP selection bias was reported in another study,^26^ which dealt with the issue in a similar way to our study, by following a kernel matching approach to select a set of nonbeneficiary BFP observations within the CIDACS 100 Million Brazilian Cohort. This method enabled us to balance groups by observable characteristics. The external validity of the study was affected by the population choice given that we considered only 1 child per mother. BFP is a binary variable in our study, and this proposal did not investigate nuances related to the value received and poverty levels. Another limitation of this study is the bias related to unmeasured confounding. Important unmeasured factors should be considered, particularly family income, which could not be included in this study. Moreover, we were unable to investigate the distribution of some established biological risk factors associated with LBW and SGA, including chronic diseases, gestational weight gain, prepregnancy body mass index, smoking, and drug use among BFP and non-BFP groups. Another important limitation of our study is that we exclusively focused on live births. Consequently, stillbirths and spontaneous abortions were not taken into consideration. Nevertheless, it is plausible that outcomes associated with these factors are attenuated when analyzing the association of BFP with birth weight indicators in more homogeneous subgroups.

## Conclusions

This cohort study found that BFP participation was associated with improved birth weight indicators. The magnitude of the improvement was greater in higher-risk groups. These findings contribute to the scope of literature evaluating integrative policies and highlighting the importance of maintaining financial support for high-risk mothers. We emphasize the importance of reducing barriers to access and use of health services. Future studies may also assess the quality of prenatal care provided to socioeconomically high-risk populations. We also highlight the importance of evaluating the association between BFP participation and the occurrence of stillbirths, abortions, and infant survival. We highlight that our evidence is associative. However, our contribution is robust and adds data to literature on the association of CCTs with maternal-child health.

## Notes

Supplement 1. **eAppendix 1.** Detailed Information: Eligible Study Population **eFigure 1.** Eligibility Criteria Applied to Obtain Initial Study Population **eAppendix 2.** *Bolsa Família* Program Characteristics **eAppendix 3.** Database Characteristics and Linkage Quality **eFigure 2.** Receiver Operating Characteristic Curve of 100 Million Brazilian Cohort and Live Birth Information System (2001-2015) Linkage: Approach 1 **eFigure 3.** Receiver Operating Characteristic Curve of 100 Million Brazilian Cohort and Live Birth Information System (2001-2015) Linkage: Approach 2 **eAppendix 4.** Missing Data **eTable 1.** Missing Data for Propensity Score Variables for Total Population **eTable 2.** Distribution of Missing Data **eTable 3.** Description of Study Population for Entire Period (2004-2015) in Accordance With Missing Data Pattern **eTable 4.** Crude Odds Ratio of Propensity Score Variables With Missing Data Category **eTable 5.** Adjusted and Weighted Coefficients Considering Propensity Score Variables With Missing Data **eAppendix 5.** Propensity Score **eTable 6.** Variables Used in Study **eFigure 4.** Common Support Area of Exposed Over Unexposed Group **eTable 7.** Propensity Score Description in Accordance With Confounding Covariate **eAppendix 6.** Adjusted Risk Ratio With δ Method **eTable 8.** Adjusted Risk Ratio With δ Method of *Bolsa Família* Beneficiaries on Birth Weight Indicators **eTable 9.** Adjusted Risk Ratio With δ Method of *Bolsa Família* Beneficiaries on Birth Weight Indicators in Accordance With Subgroup Analysis **eAppendix 7.** Analysis of Robustness for Propensity Score–Based Methods **eTable 10.** Coefficients of Adjusted and Weighted Logistic and Linear Regressions of *Bolsa Família* Beneficiaries on Birth Weight Indicators **eAppendix 8.** Subgroup Analysis **eTable 11.** Variables Used in Subgroup Analysis **eTable 12.** *Bolsa Família* and Birth Weight Indicators of the Population of Second Live Births **eTable 13.** Adjusted and Weighted Coefficients of *Bolsa Família* Beneficiaries on Birth Weight Indicators Considering Propensity Score Quintiles **eAppendix 9.** Bivariate Analyses **eTable 14.** Crude Analysis for the Association of *Bolsa Família*, Level of Education, and Race With Appointments
eReferences.

Supplement 2.
Data Sharing Statement
