# Supplementary material for: Participation in Conditional Cash Transfer Program During Pregnancy and Birth Weight–Related Outcomes
Source: JAMA Netw Open. 2023 Nov 28;6(11):e2344691. doi: 10.1001/jamanetworkopen.2023.44691 (PMC10685879; doi:10.1001/jamanetworkopen.2023.44691)
Supplement: Supplement 1. — eAppendix 1. Detailed Information: Eligible Study Population eFigure 1. Eligibility Criteria Applied to Obtain Initial Study Population eAppendix 2. Bolsa Família Program Characteristics eAppendix 3. Database Characteristics and Linkage Quality eFigure 2. Receiver Operating Characteristic Curve of 100 Million Brazilian Cohort and Live Birth Information System (2001-2015) Linkage: Approach 1 eFigure 3. Receiver Operating Characteristic Curve of 100 Million Brazilian Cohort and Live Birth Information System (2001-2015) Linkage: Approach 2 eAppendix 4. Missing Data eTable 1. Missing Data for Propensity Score Variables for Total Population eTable 2. Distribution of Missing Data eTable 3. Description of Study Population for Entire Period (2004-2015) in Accordance With Missing Data Pattern eTable 4. Crude Odds Ratio of Propensity Score Variables With Missing Data Category eTable 5. Adjusted and Weighted Coefficients Considering Propensity Score Variables With Missing Data eAppendix 5. Propensity Score eTable 6. Variables Used in Study eFigure 4. Common Support Area of Exposed Over Unexposed Group eTable 7. Propensity Score Description in Accordance With Confounding Covariate eAppendix 6. Adjusted Risk Ratio With δ Method eTable 8. Adjusted Risk Ratio With δ Method of Bolsa Família Beneficiaries on Birth Weight Indicators eTable 9. Adjusted Risk Ratio With δ Method of Bolsa Família Beneficiaries on Birth Weight Indicators in Accordance With Subgroup Analysis eAppendix 7. Analysis of Robustness for Propensity Score–Based Methods eTable 10. Coefficients of Adjusted and Weighted Logistic and Linear Regressions of Bolsa Família Beneficiaries on Birth Weight Indicators eAppendix 8. Subgroup Analysis eTable 11. Variables Used in Subgroup Analysis eTable 12. Bolsa Família and Birth Weight Indicators of the Population of Second Live Births eTable 13. Adjusted and Weighted Coefficients of Bolsa Família Beneficiaries on Birth Weight Indicators Considering Propensity Score Quintiles [file jamanetwopen-e2344691-s001.pdf]

Supplemental Online Content

Falcão IR, Ribeiro-Silva RC, Fiaccone RL, et al. Participation in conditional cash transfer program during pregnancy and birth weight–related outcomes. *JAMA Netw Open.* 2023;6(11):e2344691. doi:10.1001/jamanetworkopen.2023.44691

eAppendix 1. Detailed Information: Eligible Study Population

eFigure 1. Eligibility Criteria Applied to Obtain Initial Study Population

eAppendix 2. *Bolsa Família* Program Characteristics

eAppendix 3. Database Characteristics and Linkage Quality

eFigure 2. Receiver Operating Characteristic Curve of 100 Million Brazilian Cohort and Live Birth Information System (2001-2015) Linkage: Approach 1

eFigure 3. Receiver Operating Characteristic Curve of 100 Million Brazilian Cohort and Live Birth Information System (2001-2015) Linkage: Approach 2

eAppendix 4. Missing Data

eTable 1. Missing Data for Propensity Score Variables for Total Population

eTable 2. Distribution of Missing Data

eTable 3. Description of Study Population for Entire Period (2004-2015) in Accordance With Missing Data Pattern

eTable 4. Crude Odds Ratio of Propensity Score Variables With Missing Data Category

eTable 5. Adjusted and Weighted Coefficients Considering Propensity Score Variables With Missing Data

© 2023 Falcão IR et al. *JAMA Network Open.*

eAppendix 5. Propensity Score

eTable 6. Variables Used in Study

eFigure 4. Common Support Area of Exposed Over Unexposed Group

eTable 7. Propensity Score Description in Accordance With Confounding Covariate

eAppendix 6. Adjusted Risk Ratio With δ Method

eTable 8. Adjusted Risk Ratio With δ Method of *Bolsa Família* Beneficiaries on Birth Weight Indicators

eTable 9. Adjusted Risk Ratio With δ Method of *Bolsa Família* Beneficiaries on Birth Weight Indicators in Accordance With Subgroup Analysis

eAppendix 7. Analysis of Robustness for Propensity Score–Based Methods

eTable 10. Coefficients of Adjusted and Weighted Logistic and Linear Regressions of *Bolsa Família* Beneficiaries on Birth Weight Indicators

eAppendix 8. Subgroup Analysis

eTable 11. Variables Used in Subgroup Analysis

eTable 12. *Bolsa Família* and Birth Weight Indicators of the Population of Second Live Births

eTable 13. Adjusted and Weighted Coefficients of *Bolsa Família* Beneficiaries on Birth Weight Indicators Considering Propensity Score Quintiles

eAppendix 9. Bivariate Analyses

eTable 14. Crude Analysis for the Association of *Bolsa Família*, Level of Education, and Race With Appointments

eReferences.

This supplemental material has been provided by the authors to give readers additional information about their work.

© 2023 Falcão IR et al. *JAMA Network Open.*

eAppendix 1. DETAILED INFORMATION: ELIGIBLE STUDY POPULATION

eFigure 1. Eligibility criteria applied to obtain the initial study population.

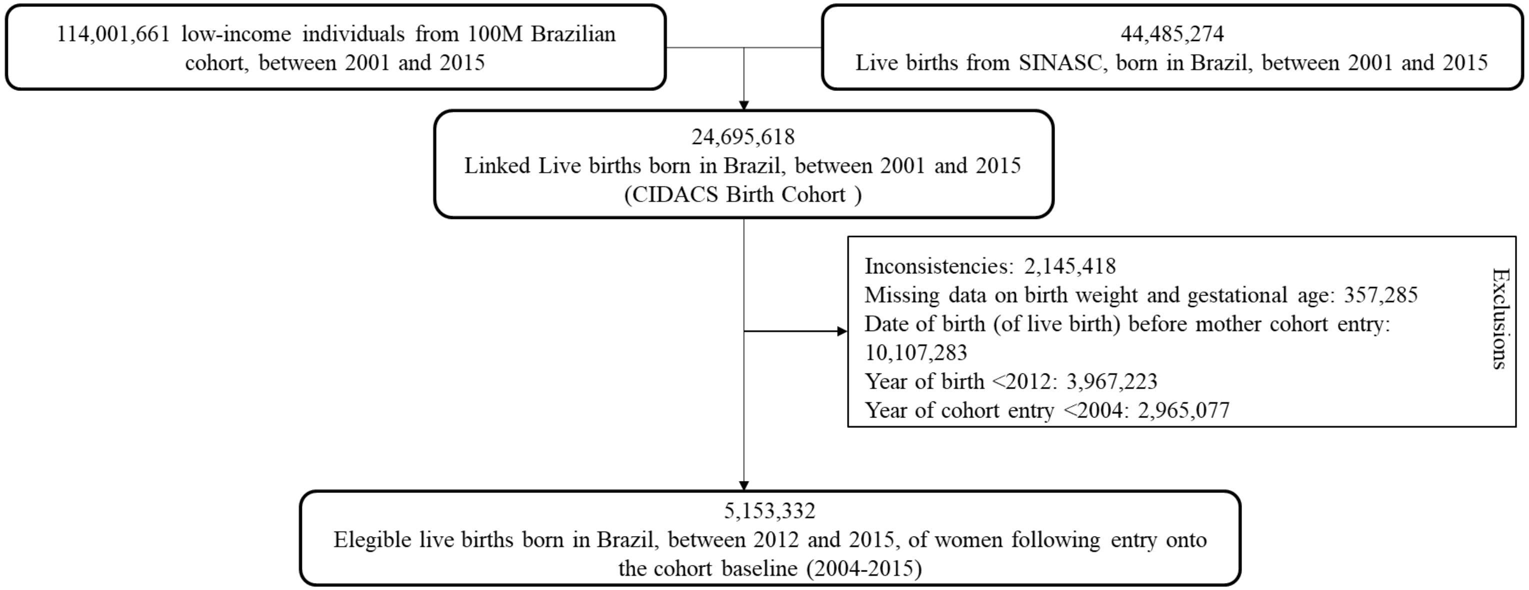

© 2023 Falcão IR et al. *JAMA Network Open.*

eAppendix 2. BOLSA FAMILIA (BFP) PROGRAM CHARACTERISTICS

First, for a family to become a Bolsa Familia (BFP) beneficiary, it must be registered on CadÚnico (Brazilian national social program register). A family with a total income of less than three minimum monthly salaries (BRL3,636.00, equivalent to USD909.00 in 2022) is considered eligible. The estimate of low-income families in each municipality is calculated based on National Household Sample Survey (PNAD) (1) data, which is used as a guide to implement BFP, but is not a ceiling, or threshold, for expenditure.

eAppendix 3. DATABASE CHARACTERISTICS AND LINKAGE QUALITY (DATA AND FIGURES PROVIDED BY THE CIDACS DATA PRODUCTION CENTER)

The cohort database comprises records containing the socio-economic data of 114,008,179 low-income individuals who have applied for social assistance programs through CadÚnico. This is the largest cohort in the world, representing approximately 55% of the entire Brazilian population (2). In addition to its cohort size, the wealth of socio-economic variables linked with health databases enables the study of social inequalities in health and the examination of specific population groups that are underrepresented in scientific research.

Data linkage

100 Million Brazilian Cohort (100M Cohort) data (2001-2015) was linked with BFP payment records (2004-2015) and SINASC (2001-2015). The cohort linkage with BFP was conducted deterministically, through the correspondence key in common with the two databases (social identification number). Record linkage (CIDACS-RL) (3, 4) was then used to link 100M Cohort (2001-2015) information with SINASC (2001-2015). CIDACS-RL is a linkage tool of individual records in two stages, using identifiers. In this case, the mother's name, age, or date of birth, and municipality of residence (3, 4) were used. The first stage

© 2023 Falcão IR et al. *JAMA Network Open.*

was the deterministic linkage of five variables, and the second based on a similarity index generated from these (4). For a linkage accuracy analysis, manual verification of a random sample of 2,000 selected records was conducted and evaluated through a receiver operating characteristic curve, from the sensitivity and specificity indexes (eFigures 2 and 3). All the linkage procedures were carried out at the Center for Data and Knowledge Integration for Health (CIDACS), Fiocruz (5), in a strict data protection environment, and in compliance with ethical and legal standards (6).

Since the mother's date of birth was only introduced to SINASC in 2011, for linkage with those born before this period, or when this date was missing, the mother's age was used. Thus, two linkage approaches are considered: 1. Using age, and 2. Using the mother's date of birth.

Summary of the databases used in the linkages:

- Indexed database (the largest): 100 million cohort; period 2001-2015; number of records 114,008,317.
- Search database (smaller database): SINASC; period 2001-2015; number of records 44,485,274.
- Variables used: Mother's name, mother's date of birth, or age, and municipality of residence.
- Number of linked records (over the defined cut-off point): 24,695,622 (55.51%); refers to the sum of the linked data for approaches 1 and 2.

© 2023 Falcão IR et al. *JAMA Network Open.*

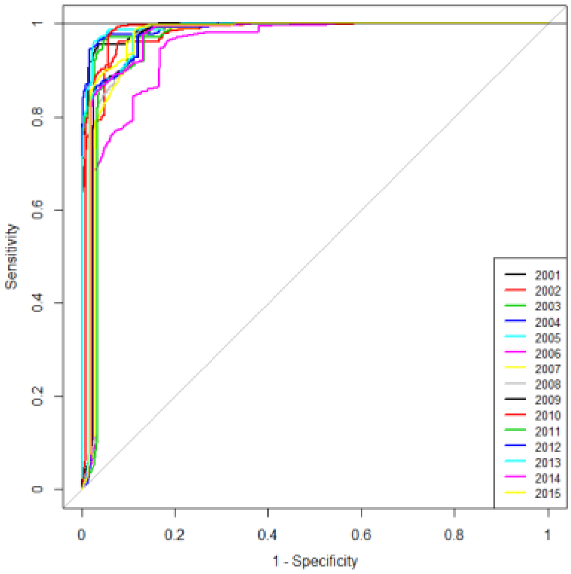

eFigure 2. ROC curve of the 100 Million Brazilian Cohort and SINASC (2001-2015) linkage – approach 1
Source: Prepared by the CIDACS Data Production Center.

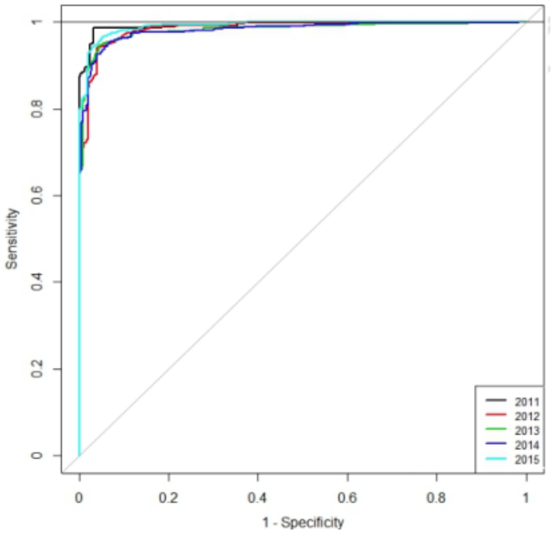

eFigure 3. ROC curve of the 100 Million Brazilian Cohort and SINASC (2011-2015) linkage – approach 2
Source: Prepared by the CIDACS Data Production Center.

© 2023 Falcão IR et al. *JAMA Network Open.*

eAppendix 4. MISSING DATA

A descriptive analysis of missing data is provided below. eTable 1 presents the percentage of missing data for each PS variable.

eTable 1. Missing data for propensity score variables for the total population (N= 4,973,146), 2004 to 2015.

Propensity score variables	Missing data		
	Overall N (%)	Non-BFP[a] N (%)	BFP[b] N (%)
State of residence	0 (0.0)	0 (0.0)	0 (0.0)
Level of education	84,183 (1.7)	24,849 (1.4)	59,334 (1.8)
Race	266,506 (5.4)	128,568 (7.3)	137,938 (4.3)
Marital status	59,659 (1.2)	17,801 (1.0)	41,858 (1.3)
Area of residency	145,109 (2.9)	71,802 (4.1)	73,307 (2.3)
Construction materials	199,709 (4.0)	100,908 (5.7)	98,801 (3.1)
Water supply	199,653 (4.0)	100,909 (5.7)	98,744 (3.1)
Electricity	199,537 (4.0)	100,871 (5.7)	98,666 (3.1)
Waste collection	199,682 (4.0)	100,909 (5.7)	98,773 (3.1)
Sanitation system	242,669 (4.9)	118,799 (6.7)	123,870 (3.9)
Overcrowding	352,391 (7.1)	158,870 (9.0)	193,521 (6.0)
Year of entry	0 (0.0)	0 (0.0)	0 (0.0)

[a] Non-BFP = Non Bolsa Familia beneficiaries: 1,766,108 individuals.
[b] BFP = Bolsa Familia beneficiaries: 3,207,038 individuals.

A descriptive analysis in accordance with the missing pattern is provided below (eTables 2 and 3). The variables "state of residence" and "year of entry" do not have a missing pattern and, therefore, was not included in the evaluation.

eTable 2. Distribution of missing data, in accordance with the patterns[a], Brazil, 2004 to 2015.

Missing patterns	N (%)
Completeness	4,277,523 (86.0)
Assorted pattern	695,623 (14.0)
Monotonicity	0 (0.0)
Total	4,973,146 (100.0)

[a] Completeness: all of the variables with information; Assorted pattern: a minimum of one variable with missing data; Monotonicity: all of the incomplete variables.

eTable 3. Description of the study population for the entire period (2004-2015), in accordance with the missing data pattern (assorted missing data) (N=695,623) and receipt of Bolsa Familia, Brazil.

Propensity score variables	Overall	Non-BFP	BFP

	%	%	%
Sociodemographic characteristics			
**Level of education**			
>=8 years of education	70.7	79.7	63.9
4-7 years	0.7	0.4	0.9
<=3 years	28.6	20.0	35.1
**Race**			
Asian	0.4	0.5	0.4
Black	8.0	7.1	8.6
Parda	63.9	58.5	67.2
Indigenous	1.2	0.6	1.6
White	26.3	33.2	22.2
**Marital status**			
Partner	54.8	57.3	53.0
No partner	45.2	42.7	47.0
Housing characteristics			
**Area of residency**			
Urban	73.8	79.8	69.9
Rural	26.2	20.2	30.1
**Construction materials**			
Brick	69.1	75.5	65.2
Wood, or other	30.9	24.5	34.8
**Water supply**			
Public network	67.2	75.0	62.4
Well, or other	32.8	25.0	37.6
**Electricity**			
Home with electricity meter	82.5	88.8	78.7
Home without meter	17.5	11.2	21.3
**Waste collection**			
Collected	67.6	76.0	62.5
Burned, buried, or other	32.4	24.0	37.5
**Sanitation system**			
Public network	45.5	54.8	39.9
Septic tank, or other	54.5	45.2	60.1
**Overcrowding**			
No (<=2 inhabitants/room)	90.9	95.0	88.4
Yes (>2 inhabitants/room)	9.1	5.0	11.6

© 2023 Falcão IR et al. *JAMA Network Open.*

To assess whether the presence of missing data is associated (or not) with neither the outcome nor the intervention, we recoded each PS variable as '1= missing value' and '0= with data'. eTable 4 presents the results of logistic regression analysis to evaluate the association between each PS variable with the missing category and: 1. BFP; 2. LBW and SGA.

eTable 4. Crude Odds Ratio of Propensity Score variables with missing data category and Bolsa Familia Beneficiaries and birth weight indicators of children born in Brazil between 2012 and 2015.

Propensity score variables/ Outcome[a]/Estimate	BFP[b]	LBW[b]	SGA[c]
	OR (95% CI)	OR (95% CI)	OR (95% CI)
Level of education	1.32 (1.30; 1.34)	0.99 (0.96; 1.01)	1.08 (1.05; 1.11)
Race	0.57 (0.57; 0.58)	0.95 (0.94; 0.97)	0.94 (0.93; 0.96)
Marital status	1.30 (1.28; 1.32)	0.96 (0.92; 0.99)	1.09 (1.05; 1.12)
Area of residency	0.55 (0.55; 0.56)	0.92 (0.90; 0.94)	0.96 (0.94; 0.98)
Construction materials	0.52 (0.52; 0.53)	0.92 (0.90; 0.94)	0.97 (0.95; 0.99)
Water supply	0.52 (0.52; 0.53)	0.92 (0.90; 0.94)	0.97 (0.95; 0.99)
Electricity	0.52 (0.52; 0.53)	0.92 (0.91; 0.94)	0.97 (0.95; 0.99)
Waste collection	0.52 (0.52; 0.53)	0.92 (0.90; 0.94)	0.97 (0.95; 0.99)
Sanitation system	0.56 (0.56; 0.57)	0.92 (0.90; 0.93)	1.01 (0.99; 1.02)
Overcrowding	0.65 (0.64; 0.65)	0.94 (0.93; 0.95)	0.95 (0.94; 0.96)

[a]BFP= Bolsa Familia Program beneficiaries; SGA=Small for gestational age; LBW= Low birthweight.

[b]Total population: 4,973,146 individuals

[c]Total population: 4,036,689 individuals

We recoded each PS variable, incorporating a category '7= missing data' to account for missing data in PS estimation. The propensity score was estimated, including the missing data category, and analytical procedures (kernel matching and weighted logistic regression) were performed for each outcome. The results can be found in eTable 5.

eTable 5. Adjusted and Weighted coefficients of Bolsa Familia beneficiaries on birth weight indicators of children born in Brazil between 2012 and 2015, considering propensity score variables with missing data category.

Outcome[a]/Estimate	Overall	Robust Standard Error	p-value	N
**K-Weighting[b] (95% CI)**				
Model 1: Low birthweight (OR)	0.89 (0.88; 0.90)	0.004	0.000	4,918,155

© 2023 Falcão IR et al. *JAMA Network Open.*

Model 2: SGA (OR)	0.99 (0.98; 1.00)	0.005	0.262	4,021,178
Model 3: Birth weight[c] (β)	17.30 (16.15; 18.46)	0.589	0.000	4,918,155

[a] SGA=Small for gestational age; non-low birthweight: birthweight ≥2500g and <4000g; low birthweight: <2500g.
[b] Logistic regression results: analysis weighted and adjusted for gestational age, sex of the live birth, mother's age at birth, and type of delivery.
[c] Linear regression results: analysis weighted and adjusted for gestational age, sex of the live birth, mother's age at birth, and type of delivery.

eAppendix 5. PROPENSITY SCORE

The propensity score (PS) was characterized as the probability of being a beneficiary (or not) of the BFP, conditioned on the baseline characteristics observed (potential confounders) (7). These methods differ from the others, since they avoid multidimensionality and can be implemented using a control variable, which is the propensity score (8). Our analysis involved a PS estimate through a logistic model, to estimate the probability of receiving BFP based on maternal variables: state of residence, urban/rural residency, level of education, race, marital status, electrical energy, water supply, sanitation system, waste collection, household density (overcrowding), and year of entry (eTable 6).

eTable 6. Variables used in the study, Brazil, 2004 to 2015.

Variable	Classification	Database
**Variables used to calculate the propensity score**		
Sociodemographic characteristics		
State of residence	27 categories related to the Brazilian federative units (26 states and 1 federal district)	100 million cohort (Cadastro Único data)
Self-reported race	Asian, black, Parda, indigenous and white	
Level of education	≥8 years of education; 4-7 years; ≤3 years	SINASC
Marital status	Partner; no partner	
Housing characteristics		
Area of residence	Urban or rural	100 million cohort (Cadastro Único data)
Construction materials	Brick, wood, or other	
Water supply	Public network, well, or other	

© 2023 Falcão IR et al. *JAMA Network Open.*

Electricity	Housing with an electricity meter; Housing without a meter	
Waste collection	Collected, burned, buried, or other	
Sanitary system	Public network, septic tank, or other	
Overcrowding	No (≤2 inhabitants/room); Yes (>2 inhabitants/room)	
Year of entry	2004/2005; 2006/2007; 2008/2009; 2010/2011; 2012/2015	
**Variables used in the adjusted models**		
Gestational age	Continuous: Gestational age in weeks<br>Categorized: <37 weeks; ≥37 weeks	
Sex of the live birth	Category: Male or female	
Mother's age on delivery	Continuous: Mother's age on delivery in years (10-49)<br>Categorized: <20 years old; 20-34 years old; ≥35 years old	SINASC
Type of delivery	Vaginal or c-section	

The beneficiaries and non-beneficiaries' individual weights were estimated from the PS through kernel-based matching (9). Kernel matching is nonparametric matching estimators which use the weighted averages of almost all of the individuals in the control group, to compile the counterfactual outcome. The weights depend on the distance between each individual in the control group, and participant observation for which the counterfactual outcome is estimated (9). Considering that the symmetrical, non-negative, and unimodal weights were estimated, greater weight is then attributed to those closest in terms of the propensity score of an assisted individual, and a lower weight on more distant observations. A major advantage of this approach is the lower variance achieved, since more information is used (9). The common support graph and descriptive analysis of the PS can be found in eFigure4 and eTable 7.

© 2023 Falcão IR et al. *JAMA Network Open.*

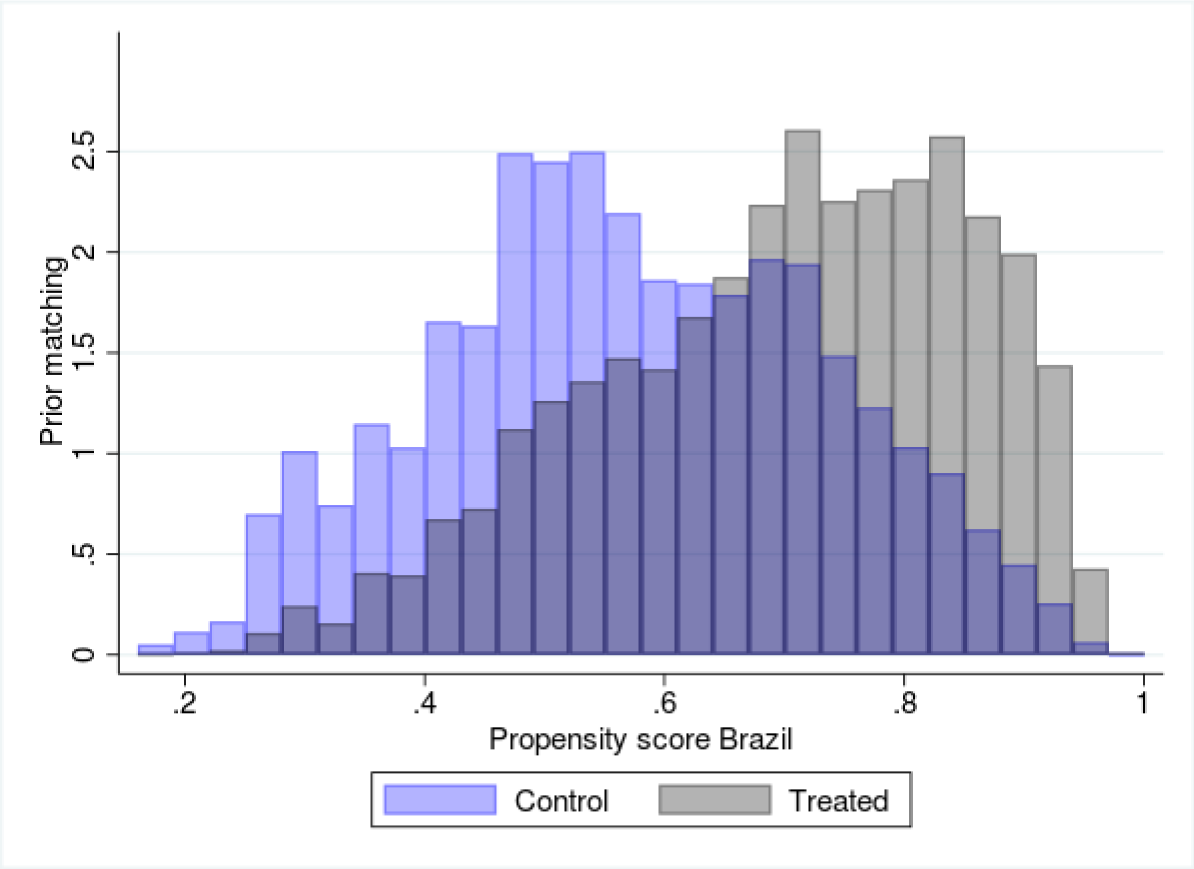

eFigure 4. Common support area of the exposed over the unexposed group, Brazil, 2004 to 2015.

© 2023 Falcão IR et al. *JAMA Network Open.*

eTable 7. Propensity score description in accordance with the confounding covariates observed, Brazil, 2004 to 2015.

Propensity score	Brazil	
	**Non-BFP**	**BFP**
Average	0.57	0.70
Deviation population	0.16	0.16
Median	0.56	0.72
Minimum	0.16	0.16
Maximum	0.98	0.99
25th Percentile	0.46	0.59
75th Percentile	0.70	0.82

eAppendix 6. ADJUSTED RISK RATIO WITH DELTA-METHOD

We estimated the adjusted risk ratio with delta-method (10) standard errors (eTables 8 and 9) after running the logistic regression models (Tables 3 and 4 of the article). The RR estimates were very close to the OR estimated by the logistic regression models.

eTable 8. Adjusted Risk Ratio with delta-method of Bolsa Familia Beneficiaries on birth weight indicators of children born in Brazil between 2012 and 2015.

Outcome[a]/Estimate	Adjusted Risk Ratio[b] (95% CI)	Robust Standard Error	p-value	N
Model 1: Low birthweight	0.91 (0.90; 0.92)	0.004	0.000	4,232,863
Model 2: SGA	0.99 (0.98; 1.00)	0.005	0.082	3,464,938

[a] SGA=Small for gestational age; non-low birthweight: birthweight ≥2500g and <4000g; low birthweight: <2500g.

[b] The analytical steps (propensity score estimation, kernel matching, weighted logistic regression, and adjusted Risk Ratio with delta-method standard errors) were conducted separately within each model. Analysis weighted and adjusted for gestational age, sex of the live birth, mother's age at birth, and type of delivery.

© 2023 Falcão IR et al. *JAMA Network Open.*

eTable 9. Adjusted Risk Ratio with delta-method of Bolsa Familia Beneficiaries on birth weight indicators of children born in Brazil between 2012 and 2015, in accordance with subgroup analysis.

Subgroup[a]/ Outcome[b]/Estimate	LBW (OR)				SGA (OR)			
	Adjusted Risk Ratio[c] (95% CI)	Robust Standard Error	p-value	N	Adjusted Risk Ratio[c] (95% CI)	Robust Standard Error	p-value	N
**Prenatal appointments**								
Model 7: Appointments ≥7	0.93 (0.92; 0.95)	0.007	0.000	2,542,051	1.02 (1.00; 1.03)	0.006	0.008	2,089,585
Model 8: Appointments <7	0.89 (0.88; 0.90)	0.005	0.000	1,601,658	0.97 (0.96; 0.99)	0.007	0.001	1,299,972
**Self-reported maternal race**								
Model 9: Asian	0.87 (0.74; 1.02)	0.072	0.081	14,780	0.99 (0.84; 1.17)	0.082	0.918	12,088
Model 10: Black	0.89 (0.86; 0.92)	0.013	0.000	329,995	0.95 (0.92; 0.98)	0.015	0.001	272,129
Model 11: Parda	0.91 (0.90; 0.92)	0.006	0.000	2,566,408	0.99 (0.98; 1.00)	0.006	0.083	2,087,464
Model 12: Indigenous	0.76 (0.65; 0.89)	0.060	0.001	28,369	0.81 (0.70; 0.93)	0.058	0.003	22,258
Model 13: White	0.94 (0.93; 0.96)	0.007	0.000	1,293,632	1.03 (1.02; 1.05)	0.008	0.000	1,071,086
**Maternal level of education**								
Model 14: ≥8 years of education	0.93 (0.92; 0.94)	0.005	0.000	2,913,202	1.03 (1.02; 1.04)	0.005	0.000	2,402,501
Model 15: 4-7 years	0.90 (0.89; 0.92)	0.008	0.000	1,120,062	0.96 (0.95; 0.98)	0.009	0.000	906,413
Model 16: ≤3 years	0.80 (0.76; 0.84)	0.020	0.000	199,483	0.85 (0.81; 0.89)	0.021	0.000	155,603

[a] All models were conducted separately within each category.

[b] SGA=Small for gestational age; non-low birthweight: birthweight ≥2500g and <4000g; LBW= Low birthweight (<2500g).

[c] The analytical steps (propensity score estimation, kernel matching, weighted logistic regression, and adjusted Risk Ratio with delta-method standard errors) were conducted separately within each level of education, self-reported maternal race, and appointments. Analysis weighted and adjusted for gestational age, sex of the live birth, mother's age at birth, and type of delivery.

© 2023 Falcão IR et al. *JAMA Network Open.*

eAppendix 7. ANALYSIS OF ROBUSTNESS FOR PROPENSITY SCORE-BASED METHODS

We used inverse probability of treatment weighting (IPTW) as an alternative approach to estimate the association between BFP participation and birth weight indicators. The analysis steps comprise PS and weight estimations, and weighted logistic regression. Logistic models were employed, with non-beneficiaries assigned weights of E(PS)/(1-E(PS)), and beneficiaries assigned weights of 1 (11). The final estimates were accessed through weighted and adjusted regression models (the same adjustment variables used in the main analysis).

The results of the inverse probability of treatment weighting (IPTW) are presented below (eTable10). The results for regression models adjusted and weighted for IPTW and Kernel were very close.

eTable 10. Coefficients of adjusted and weighted logistic and linear regressions of Bolsa Familia Beneficiaries on birth weight indicators of children born in Brazil between 2012 and 2015.

Outcome[a]/Estimate	Overall	Robust Standard Error	p-value	N
**IPTW[b] (95% CI)**				
Model 1: Low birthweight (OR)	0.89 (0.88; 0.90)	0.005	0.000	4,240,288
Model 2: SGA (OR)	0.98 (0.97; 0.99)	0.005	0.003	3,471,613
Model 3: Birth weight[a] (β)	18.91 (17.68; 20.14)	0.626	0.000	4,240,288

[a] SGA=Small for gestational age; non-low birthweight: birthweight ≥2500g and <4000g; low birthweight: <2500g.
[b] Logistic regression results: analysis weighted and adjusted for gestational age, sex of the live birth, mother's age at birth, and type of delivery.
[c] Linear regression results: analysis weighted and adjusted for gestational age, sex of the live birth, mother's age at birth, and type of delivery.

eAppendix 8. SUBGROUP ANALYSIS

A similar approach was performed by subgroups. The analytical steps (propensity score estimation, kernel matching, and weighted logistic regression) were conducted separately within each level of education, self-reported maternal race, and number of prenatal appointments. In our study, we did not

© 2023 Falcão IR et al. *JAMA Network Open.*

include the level of education variable in the PS estimation for the subgroup analyses based on education level, and those based on self-reported race and ethnicity. Detailed information on the variables used in the study can be found in eTable 11.

eTable 11. Variables used in subgroups analysis.

Variable	Classification	Database
**Variables used to calculate the propensity score**		
Sociodemographic characteristics		
State of residence	27 categories related to the Brazilian federative units (26 states and 1 federal district)	100 million cohort (Cadastro Único data)
Self-reported race	Asian, black, Parda, indigenous and white	
Level of education	≥8 years of education; 4-7 years; ≤3 years	SINASC
Marital status	Partner or no partner	
Housing characteristics		
Area of residence	Urban, or rural	100 million cohort (Cadastro Único data)
Construction materials	Brick, wood, or other	
Water supply	Public network, well, or other	
Electricity	Housing with an electricity meter; Housing without a meter	
Waste collection	Collected, burned, buried, or other	
Sanitary system	Public network, septic tank, or other	
Overcrowding	No (≤2 inhabitants/room); Yes (>2 inhabitants/room)	

© 2023 Falcão IR et al. *JAMA Network Open.*

Year of entry	2004/2005; 2006/2007; 2008/2009; 2010/2011; 2012/2015	
**Variables used in the adjusted models**		
Gestational age	Continuous: Gestational age in weeks	SINASC
	Categorized: <37 weeks; ≥37 weeks	
Sex of the live birth	Category: Male or female	
Mother's age on delivery	Continuous: Mother's age on delivery in years (10-49)	
	Categorized: <20 years old; 20-34 years old; ≥35 years old	
Type of delivery	Vaginal or c-section	
**Variables used in the adjusted models (analysis for second live birth)**		
Previous child's birth weight	Continuous: previous child's birth weight in grams	Variables created from the ranking of live births by each mother
	Categorized: Low birthweight (Yes: <2500g; No: ≥2500g)	
Interbirth interval	Continuous: Interval between deliveries in months	
	Categorized: <12 months; 12-24 months; >24 months	
Previous child's prematurity	Yes: <37w; No: ≥37w	
**Variables used to classify subgroups**		

© 2023 Falcão IR et al. *JAMA Network Open.*

Number of prenatal appointments	Categorized: < median: 7 appointments; ≥ median: 7 or more appointments	SINASC
Self-reported race	Asian, black, Parda, indigenous and white	100 million cohort (Cadastro Único data)
Level of education	≥8 years of education; 4-7 years; ≤3 years	SINASC

Furthermore, we chose second births, in order to examine the association of BFP with birth outcomes among multiparous women, while also accounting for their prior pregnancy characteristics, using weighted and adjusted models (eTable 12).

In order to estimate the association of BFP with low birthweight and small for gestational age (SGA), logistic models were used, weighted and adjusted for the following risk factors (categorized variables): previous child's low birthweight, previous child's prematurity, interbirth interval, gestational age, sex of the live birth, maternal age at birth, and type of delivery. The linear model was used to estimate the effect of BFP on the birth weight (continuous), weighted and adjusted by the previous child's birth weight in grams, the interbirth interval, gestational age in weeks, and maternal age on delivery in years.

eTable 12. Bolsa Familia and birth weight indicators of the population of second live births (N=1,297,333) in Brazil between 2012 and 2015.

Outcome[a]/Estimate	K-Weighting[b] (95% CI)
Low birthweight (OR)	0.95 (0.92; 0.98)
SGA (OR)	1.04 (1.01; 1.08)
Birth weight[c] (β)	1.97 (-3.22; 3.15)

[a] SGA=Small for gestational age NLBW= Non-low birthweight; LBW= Low birthweight (<2500g).

[b] Analysis weighted and adjusted by multivariate logistic regression, including categorized variables known as risk factors for the outcomes presented (previous low birthweight, interdelivery interval, categorized gestational age, sex of the live birth, mother's categorized age, and type of delivery).

[c] Multivariate linear regression, including risk factors for the outcome presented (previous weight, interdelivery interval, gestational and mother's age, sex of the live birth, and type of delivery).

© 2023 Falcão IR et al. *JAMA Network Open.*

Additionally, we investigated the association between BFP and birth outcomes based on PS quintiles. We followed similar procedures, employing weighted and adjusted models (eTable 13). We observed estimates of greater magnitude in the 5th quintile, where PS scores are higher, indicating individuals more likely to receive the PBF based on the covariates used.

eTable 13. Adjusted and Weighted coefficients of Bolsa Familia beneficiaries on birth weight indicators of children born in Brazil between 2012 and 2015, considering propensity score (PS) quintiles.

Outcome[a]/Estimate	Estimate[b,c] (95% CI)	Robust Standard Error	p-value	N
**PS 1st Quintile**				
Model 1: Low birthweight (OR)	0.97 (0.95; 0.99)	0.010	0.003	846,083
Model 2: SGA (OR)	1.04 (1.02; 1.06)	0.010	0.000	707,656
Model 3: Birth weight[c] (β)	7.74 (5.72; 9.76)	1.031	0.000	846,083
**PS 2nd Quintile**				
Model 4: Low birthweight (OR)	0.92 (0.90; 0.94)	0.010	0.000	851,569
Model 5: SGA (OR)	1.02 (1.00; 1.04)	0.010	0.020	710,138
Model 6: Birth weight[c] (β)	10.28 (8.25; 12.31)	1.037	0.000	851,569
**PS 3rd Quintile**				
Model 7: Low birthweight (OR)	0.90 (0.88; 0.92)	0.009	0.000	847,598
Model 8: SGA (OR)	1.02 (1.00; 1.04)	0.009	0.050	694,394
Model 9: Birth weight[c] (β)	13.07 (10.89; 15.24)	1.110	0.000	847,598
**PS 4th Quintile**				
Model 10: Low birthweight (OR)	0.87 (0.85; 0.89)	0.010	0.000	843,662
Model 11: SGA (OR)	1.01 (0.99; 1.03)	0.010	0.333	682,977
Model 12: Birth weight[c] (β)	16.65 (14.14; 19.16)	1.281	0.000	843,662
**PS 5th Quintile**				
Model 13: Low birthweight (OR)	0.85 (0.82; 0.87)	0.013	0.000	843,327
Model 14: SGA (OR)	0.94 (0.91; 0.96)	0.012	0.000	669,270
Model 15: Birth weight[c] (β)	27.08 (23.65; 30.50)	1.748	0.000	843,327

[a] SGA=Small for gestational age; non-low birthweight: birthweight ≥2500g and <4000g; low birthweight: <2500g.

[b] Logistic regression results: analysis weighted and adjusted for gestational age, sex of the live birth, mother's age at birth, and type of delivery.

[c] Linear regression results: analysis weighted and adjusted for gestational age, sex of the live birth, mother's age at birth, and type of delivery.

© 2023 Falcão IR et al. *JAMA Network Open.*

eAppendix 9. BIVARIATE ANALYSES

Bivariate analyses were performed considering the number of appointments during prenatal care (reference: ≥7 consultations; <7 consultations) as the outcome (eTable 14). We used receipt of BFP, level of education, and self-reported maternal race as the exposure variables. The results indicated a higher likelihood of BFP beneficiaries, women with fewer years of education, and non-white women having fewer than 7 prenatal consultations.

eTable 14. Crude analysis for the association of Bolsa Família, level of education and self-reported race with appointments in the population of women with live births in Brazil between 2012 and 2015.

Exposure/Estimate	OR[a] (95% CI)
**Bolsa Familia**	
No	-
Yes	1.58 (1.58; 1.59)
**Level of education**	
≥8 years	-
4 to 7 years	1.64 (1.63; 1.64)
<4 years	2.01 (1.99; 2.03)
**Self-reported race**	
White	-
Asian	1.42 (1.37; 1.46)
Black	1.65 (1.63; 1.66)
Parda	1.66 (1.65; 1.67)
Indigenous	3.24 (3.17; 3.32)

[a] Crude odds ratio obtained by bivariate logistic regression.

© 2023 Falcão IR et al. *JAMA Network Open.*

eReferences

1. MDS. Manual de Gestão do Programa Bolsa Família [Single Registry Management Manual for Federal Government Social Programs]. 3 ed. Brasília: 2018; [cited 2022 September 2020 Available at: https://central3.to.gov.br/arquivo/408993/.
2. Center for Data and Knowledge Integration for Health Center for Data and Knowledge Integration for Health. Cohort of 100 million Brazilians: Centro de Integração de Dados e Conhecimentos para a Saúde; 2018 [cited 2018 December 2018]. Available at: https://cidacs.bahia.fiocruz.br/en/platform/cohort-of-100-million-brazilians/.
3. Almeida D, Gorender D, Ichihara MY, Sena S, Menezes L, Barbosa GCG, et al. Examining the quality of record linkage process using nationwide Brazilian administrative databases to build a large birth cohort. BMC Med Inform Decis Mak. 2020;20(1):173. Epub 2020/07/28. doi: 10.1186/s12911-020-01192-0. PubMed PMID: 32711532; PubMed Central PMCID: PMCPMC7382864.
4. Barbosa GCG, Ali MS, Araujo B, Reis S, Sena S, Ichihara MYT, et al. CIDACS-RL: a novel indexing search and scoring-based record linkage system for huge datasets with high accuracy and scalability. BMC Med Inform Decis Mak. 2020;20(289). doi: 10.1186/s12911-020-01285-w.
5. Barreto ML, Ichihara MY, Almeida BA, Barreto ME, Cabral L, Fiaccone RL, et al. The Center for Data and Knowledge Integration for Health (CIDACS): Linking health and social data in Brazil. Int J Popul Data Sci. 2019;4(2):1-12. doi: 10.23889/ijpds.v4i2.1140.
6. Harron K, Dibben C, Boyd J, Hjern A, Azimaee M, Barreto ML, et al. Challenges in administrative data linkage for research. BIG DATA SOC. 2017;4(2):2053951717745678. Epub 2018/11/02. doi: 10.1177/2053951717745678. PubMed PMID: 30381794; PubMed Central PMCID: PMCPMC6187070.
7. Rosenbaum PR, Rubin DB. Reducing Bias in Observational Studies Using Subclassification on the Propensity Score. J Am StatAssoc. 1984;79(387):516-24. doi: 10.2307/2288398.
8. Becker SO, Ichino A. Estimation of Average Treatment Effects Based on Propensity Scores. The Stata J. 2002;2(4):358-77. doi: 10.1177/1536867X0200200403.
9. Caliendo M, Kopeinig S. Some practical guidance for the implementation of propensity score matching. J Econ Sur. 2008;22(1):31-72. doi: https://doi.org/10.1111/j.1467-6419.2007.00527.x.
10. Norton, E. C., Miller, M. M., & Kleinman, L. C. (2013). Computing Adjusted Risk Ratios and Risk Differences in Stata. The Stata Journal, 13(3), 492–509. https://doi.org/10.1177/1536867X1301300304
11. Austin PC. The use of propensity score methods with survival or time-to-event outcomes: reporting measures of effect similar to those used in randomized experiments. Stat Med. 2014;33(7):1242-58

© 2023 Falcão IR et al. *JAMA Network Open.*
