# Supplementary material for: Participation in Conditional Cash Transfer Program During Pregnancy and Birth Weight–Related Outcomes
Source: JAMA Netw Open. 2023 Nov 28;6(11):e2344691. doi: 10.1001/jamanetworkopen.2023.44691 (PMC10685879; doi:10.1001/jamanetworkopen.2023.44691)
Supplement: Supplement 2. — Data Sharing Statement [file jamanetwopen-e2344691-s002.pdf]

Data Sharing Statement

Falcão. Participation in Conditional Cash Transfer Program During Pregnancy and Birth Weight–Related Outcomes. *JAMA Netw Open*. Published online November 28, 2023. doi:10.1001/jamanetworkopen.2023.44691

Data
Data available: Yes
Data types: Data dictionary, Other (please specify)
Additional Information: Data described in the manuscript, code book, and analytic code will be made available upon request, pending application and approval.
How to access data: ila.falcao@fiocruz.br
When available: beginning date: 01-10-2024, end date: 01-10-2025

Supporting Documents
Document types: None

Additional Information
Who can access the data: The data described in the manuscript, codebook and analytical code will be made available to anyone who requests it, pending application and approval.
Types of analyses: Any request will be evaluated upon request made to the data curation team
Mechanisms of data availability: The data can be made available after data curation team approval of a proposal, with a signed data access agreement.
Any additional restrictions: All data supporting the findings presented here were obtained from the Center for Data and Knowledge Integration for Health (CIDACS). Importantly, restrictions apply to the availability of these data, licensed for exclusive use in the current study, and are thus not publicly available. Upon reasonable request and with the express permission of CIDACS, the authors are willing to make every effort to grant data availability. No data was used or analyzed for this protocol. Data will be included in completed study.
